# Oral habits among preschool Indian children at Durg-Bhilai city

**DOI:** 10.6026/973206300200528

**Published:** 2024-05-31

**Authors:** Pratik Surana, Surya Madhavi Dinavahi, Arunkumar Sajjanar, Neha Rani Gupta, Pooja Sharma, Ritu J Sabharwal

**Affiliations:** 1 Department of Pedodontics and Preventive Dentistry, Maitri College of Dentistry and Research Centre, Durg, Chhattisgarh, India; 2 MPH, University of New Haven, West haven, Connecticut 06516, USA; 3 Department of Pediatrics and Preventive Dentistry, Swargiya Dadasaheb Kalmegh Smruti Dental College and Hospital, Nagpur, Maharashtra, India; 4 Department of Dentistry, Shri Balaji Institute of Medical Sciences, Mowa, Raipur, Chhattisgarh, India; 5 Department of Pedodontics and Preventive Dentistry, Maitri College of Dentistry and Research Center, Durg, Chhattisgarh, India; 6 Department of Pedodontics and Preventive Dentistry, Kalinga Institute of Dental Sciences, KIIT Deemed to be University, Bhubaneswar, Odisha, India

**Keywords:** Oral habits, prevalence, malocclusion

## Abstract

One of the major contributing causes to the development of malocclusion and other negative impacts on orofacial complexes is oral habits during and after preschool. Therefore, it is of interest to ascertain the prevalence of oral habits in preschoolers in Durg-Bhilai, Chhattisgarh, India. Using the cluster sampling technique, four schools two from each of Durg and Bhilai City were chosen to participate in the study. 400 LKG and UKG students, along with their mothers or caregivers, were chosen based on the inclusion/exclusion criteria. The Chi-square test was employed in the statistical study. Thus, preschoolers at durg-bhilai city, Chhattisgarh, India, had a high prevalence of oral habits.

## Background:

Habits are extremely complicated acquired routines. They begin as a conditioned reflex throughout a subject's development and maturation and are learned by doing the same or comparable acts repeatedly, which results in the development of instinctive tendencies. Oral function includes swallowing, chewing, and articulation. [1] Oral habits can be parafunctional or functional. Practice of an involuntary or non-functional action leads to the development of parafunctional oral habits. Diagnosing parafunctional behaviours is crucial because they can lead to malocclusion since they disrupt the jaws' normal growth pattern and the secondary dentition's occlusion development. [2,3] It is common to see malocclusion regardless of place of residence, ethnic group, gender, age, or social class.[4] Malocclusion is caused locally by a variety of careless oral habits, posture, and muscular activity. Dentist bear a great deal of responsibility for the diagnosis, treatment, or evaluation of dental abnormalities.[5] Environmental factors may receive significant attention; nevertheless, it is critical to acknowledge that numerous pathological determinants of malocclusion possess a genetic foundation, rendering them unavoidable.[6] Dentists can have a significant impact in this area. Early intervention in oral habits may constitute a pivotal initial step towards averting persistent occlusal issues.[7,8] Therefore, it is of interest to find out how common oral habits are in children aged three to five, given the significance of oral habits and their serious problems, and the fact that no previous research has been done in Durg-Bhilai city.

## Material and Methods:

A cross-sectional survey with community-based approach was carried out between February 1, 2024, and March 31, 2024 among kids in LKG and UKG at four randomly chosen Durg-Bhilai City schools. The Institutional Review Committee of Maitri College of Dentistry and Research Centre, Durg granted ethical approval [Ref/MCDRC/2024/Jan/09(A)]. The parents or legal guardians gave their informed consent, and the children who took part in the study gave their assent. The sample size was determined to be 400 children, accounting for a 95% confidence interval (CI=1.96), a permissible error of 14%, and an additional 10% to accommodate non-responses, based on the study methodology described by Mehdipour A *et al.* [9] Only children with parental consent present at the day of examination were included in the study whereas children with fractured tooth, medical condition and congenital anomalies i.e. cleft lip/palate were excluded. Parents and legal guardians received a questionnaire with questions on the sample's demographics, oral habits, and orthodontic treatment history. For the oral health assessment, each child was instructed to sit in a chair positioned in natural light to facilitate visibility. The examination process commenced with an extra oral examination, followed by an intraoral examination. For the intraoral examination, dental professionals utilized a straight probe and a mouth mirror (number 4) to thoroughly inspect the oral cavity for any signs of dental issues or anomalies. This meticulous approach ensured a comprehensive evaluation of the children's oral health status.

To confirm lip behaviors such as lip wetness and lip sucking in the studied children, researchers relied on a combination of factors including positive parental history and specific physical findings upon examination. The presence of a reddish, chapped area below the vermilion border of the lips served as a visual indicator of such behaviors. Additionally, an exaggerated mento-labial sulcus - the crease or fold between the lower lip and the chin - further supported the diagnosis. These criteria helped in accurately identifying children engaging in habitual lip behaviors, aiding in the study's assessment of oral health impacts related to these actions. [1, 10] The evaluation of thumb sucking was conducted through the assessment of the child's oral cavity and the proclination of the maxillary incisors. The confirmation of mouth breathing was achieved through the application of the Mirror test/Fog test, coupled with the observation of an incompetent upper lip in conjunction with inflamed marginal gingiva [1]. Positive family histories, the existence of dentition attrition - which can occasionally result in pulpal exposure- and the unusual movement of teeth during examination, were used to demonstrate the presence of bruxism. The examiner's two hands were used to palpate the masseter muscle and concurrently retract the lower lip slightly with both thumbs to reveal any tongue push that may have been present. The patients were instructed to swallow the saliva while holding their hands in this manner. Individuals who exhibited a reduction in size or nonexistence of discernible masseter muscle activity, but yet shoved their tongue forward, causing it to protrude between their incisors, were classified as tongue thrust swallowers. [1,11,12] Self-destructive oral behaviors, including the manipulation of gingiva, the biting of lips, cheeks, and tongues, as well as the forceful disruption of the frenum, were corroborated through a familial history of such practices, alongside the clinical observation of inexplicable damage to oral tissues during examination. [1] The gathered dataset was utilized to evaluate the frequency of various oral behaviors among children. The Chi-square statistical test was employed to examine distinctions between male and female subjects and their significance (p<0.05), using the Statistical Package for the Social Sciences (SPSS) software, version 25.0.

## Result:

In the study, 400 children were assessed, of whom 210 (52.5%) were identified as boys and 190 (47.5%) as girls. Among the participants aged 3 to 5 years, mean age was 4.0 ± 0.05 years. The most prevalent oral habits, according to the study's findings, were thumb sucking followed by mouth breathing lip biting, and bruxism. The overall and individual frequency of oral habits in children aged 3 to 5 who have gender disparities is shown in Table 1. Overall prevalence of oral habits in 3 to 5 year-old preschool children was found to be 38%. In the male population, a higher prevalence of oral habits was observed at 42.8%, in contrast to the female population, which demonstrated a prevalence of 32.6%; this difference was determined to be statistically significant (p<0.05). However, upon examination of specific oral habits on an individual basis, significant disparities between genders were not evident.

## Discussion:

The terms "harmful" or "parafunctional" are frequently used to describe oral injurious habits, which include thumb sucking, bottle feeding, tongue thrusting, nail biting, lip biting, and mouth breathing. These behaviours can have an impact on the body's stomatognathic system and directly impact one's quality of life. [13] The objective of the present investigation was to ascertain the frequency of oral habits among children aged 3 to 5 in Durg-Bhilai City with the aim of mitigating potential adverse effects. This study's overall oral habit prevalence of 38% is consistent with research findings from Rajchanovska and Zafirova-Ivanovska (35.9%) [14] and Dhull *et al.* (36%) [10] However, lesser prevalence of these oral habits has been reported by Shetty *et al.* (1998) [2] and Kharbanda *et al.* (2003) who found that among children from north and south India, the prevalence was 25.5% and 29.7%, respectively.[15] According to the current study, oral habits are more common in boys (42.8%) than in girls (32.6%), which is consistent with findings from a 2022 study by Rai *et al.* among Indian children. [1] Thumb sucking was found in 42% of study sample which is very high as compared to the prevalence reported by Amitha and Arun (1.9%) [16] and Kharbanda *et al.* [15] reported 0.7% prevalence. In our investigation, Bruxism emerged as the second most prevalent habit, exhibiting a prevalence rate of 19%, which was significantly higher than the findings reported by Shetty and Munshi. [2] Conversely, Dhull KS *et al.* identified a lower prevalence of Bruxism in their research.[10] Boys were more likely than girls to have all of the oral habits that were evaluated, but these disparities did not hold true when the habits were analyzed individually. Some of the drawbacks of this research include the parents' possible bias in their answers, the child's lack of cooperation, and their lack of understanding regarding oral habits. More studies in this area are advised to be conducted in the future with the goal of examining the many factors influencing the prevalence of oral habits and the difficulties associated with them across a larger age range.

## Conclusion:

The overall prevalence of harmful oral behaviours was found to be considerable in the current group, according to the data. The most common behaviour seen was thumb sucking. Information from the data used as a baseline for developing preventative measures to break the oral habits.

## Figures and Tables

**Table 1:** Aggregate and Specific Prevalence of Oral Habits

**Type of Habit**	**Boys (*n*=210)**	**Girl (*n*=190)**	**Total (*n*=400)**	**P-value**
Thumb sucking	40 (44.4%)	25 (39.7%)	65 (42.8%)	NS
Mouth breathing	15 (16.7%)	10 (15.9%)	25 (16.4%)	NS
Tongue thrusting	10 (11.1%)	7 (11.1%)	17 (11.2%)	NS
Lip biting	10 (11.1%)	6 (9.5%)	16 (10.5%)	NS
Bruxism	15 (16.7%)	14 (22.2%)	29 (19.0%)	NS
Any other	-	-	-	-
Total	90 (42.8%)	63 (32.6%)	153 (38%)	<0.05*
NS= Not Significant, *= Significant
